# Neurosecretory Protein GM–Expressing Neurons Participate in Lipid Storage and Inflammation in Newly Developed Cre Driver Male Mice

**DOI:** 10.3390/biomedicines11123230

**Published:** 2023-12-06

**Authors:** Yuki Narimatsu, Masaki Kato, Eiko Iwakoshi-Ukena, Shogo Moriwaki, Ayano Ogasawara, Megumi Furumitsu, Kazuyoshi Ukena

**Affiliations:** Laboratory of Neurometabolism, Graduate School of Integrated Sciences for Life, Hiroshima University, Higashi-Hiroshima, Hiroshima 739-8521, Japaniwakoshi@hiroshima-u.ac.jp (E.I.-U.); d221199@hiroshima-u.ac.jp (S.M.);

**Keywords:** neurosecretory protein GM, hypothalamus, neuropeptide, obesity, chronic inflammation, chemogenetics

## Abstract

Obesity induces inflammation in the hypothalamus and adipose tissue, resulting in metabolic disorders. A novel hypothalamic neuropeptide, neurosecretory protein GM (NPGM), was previously identified in the hypothalamus of vertebrates. While NPGM plays an important role in lipid metabolism in chicks, its metabolic regulatory effects in mammals remain unclear. In this study, a novel Cre driver line, NPGM-Cre, was generated for cell-specific manipulation. Cre-dependent overexpression of *Npgm* led to fat accumulation without increased food consumption in male NPGM-Cre mice. Chemogenetic activation of NPGM neurons in the hypothalamus acutely promoted feeding behavior and chronically resulted in a transient increase in body mass gain. Furthermore, the ablated NPGM neurons exhibited a tendency to be glucose intolerant, with infiltration of proinflammatory macrophages into the adipose tissue. These results suggest that NPGM neurons may regulate lipid storage and inflammatory responses, thereby maintaining glucose homeostasis.

## 1. Introduction

Obesity is recognized as one of the risk factors associated with metabolic abnormalities, including type 2 diabetes, hypertension, hyperlipidemia, and cardiovascular disease [1,2,3,4]. Recent studies have highlighted the important role of chronic inflammation in adipose tissue in the development of metabolic abnormalities [5,6,7,8]. Several reports have demonstrated the involvement of neuropeptides and their producing neurons within the hypothalamus in feeding behavior, obesity, chronic inflammation, and related diseases. There is accumulating evidence regarding the arcuate nucleus (Arc). Neuropeptide Y (NPY) and agouti-related protein (AgRP) exhibit potent orexigenic effects [9]. The intracerebroventricular (i.c.v.) injection of AgRP has been shown to upregulate the mRNA expressions of tumor necrosis factor-α (TNF-α), a proinflammatory cytokine, in epididymal white adipose tissue (eWAT) via the sympathetic nervous system (SNS) [10]. AgRP-expressing neurons are also involved in energy homeostasis [11,12]. Conversely, α-melanocyte-stimulating hormone (α-MSH), derived from proopiomelanocortin (POMC), contributes to anorexigenic behavior in rodents through melanocortin receptor type 4 (MC4R) [13,14]. Chronic inhibition of the POMC- and MC4R-expressing neurons results in profound obesity [15]. In addition to the Arc, steroidogenic factor 1 (SF1) neurons, which are vital in regulating energy homeostasis in the ventromedial hypothalamus (VMH), have been found to inhibit inflammatory responses in the inguinal WAT (iWAT) of mice fed a high-fat diet (HFD) [16]. Furthermore, the activation of corticotropin-releasing hormone (CRH)-expressing neurons in the paraventricular nucleus of the hypothalamus (PVH) shifts the preference from a carbohydrate-rich diet to an HFD, and these neurons are involved in the development of HFD-induced obesity [17,18]. Despite accumulating evidence on the hypothalamic regulation of energy homeostasis as described above, the complete picture remains complex and elusive.

Two novel genes, *Fam237a*/*Gm39653* and *Fam237b*/*Gm8773*, encoding precursor proteins have been identified in the hypothalamus of chickens, rats, mice, and humans [19,20,21,22]. Owing to their distinct C-terminal amino acids, mature proteins derived from these precursor proteins are termed neurosecretory protein GL (NPGL) and neurosecretory protein GM [19]. To date, the essential role of NPGL in energy metabolism has been revealed. NPGL-expressing cells are localized in the lateroposterior part of the Arc (ArcLP) [21]. An acute i.c.v. injection of NPGL has been shown to stimulate feeding behavior [21]. Hypothalamic overexpression of *Npgl* leads to obesity in mice fed normal chow (NC) and high-calorie diets [23]. As for NPGM, we have reported that NPGM and NPGL are co-localized in the medial mammillary nucleus within the hypothalamic infundibulum of chicken [24]. We also found that NPGM accelerated lipid deposition without overeating in chicks [25]. A recent report showed that NPGM-enriched neurons are GABAergic and represent a subpopulation of AgRP-expressing neurons in mice [26]. Furthermore, recent findings revealed that an acute i.c.v. injection of NPGM stimulated feeding behavior in mice [27]. These data indicate that NPGM and/or NPGM-expressing neurons are key hypothalamic regulators of energy metabolism in addition to NPGL. Nevertheless, little is known about the effects of NPGM and NPGM neurons on energy metabolism, primarily due to the yet-to-be-elucidated intramolecular disulfide bond pattern of mammalian endogenous NPGM.

In this study, new Cre-driver mice were generated that bicistronically express NPGM and Cre recombinase, and neuron-specific analyses were conducted to provide further insight into the hypothalamic regulation of energy metabolism by NPGM. The present study analyzes the Cre-dependent overexpression of the precursor gene, chemogenetic manipulation, and neural ablation of NPGM neurons, with a focus on the effects of NPGM neurons on feeding behavior, fat accumulation, and chronic inflammation in the WAT.

## 2. Materials and Methods

### 2.1. Animals

NPGM-Cre mice were generated using the CRISPR-Cas9 system (Cyagen Biosciences, Inc., Santa Clara, CA, USA). The gRNAs designed for the CRISPR target sequence (Forward: 5′-AATGGCAGGACGTGATCTGAAGG-3′, Reverse: 5′-CACGTCCTGCCATTTTCTGTGGG-3′) and the donor vector for integration of the P2A (a self-cleaving peptide)-Cre sequence, along with Cas9 mRNA, were co-injected into fertilized mouse eggs to generate targeted knock-in offspring. Additionally, a synonymous mutation, S124 (TCC to AGT), was introduced to prevent sequence binding and recutting by gRNA after homology-directed repair. The desired mutation in the F0 founder mice was identified through PCR, followed by sequence analysis. Germline transmission and generation of F1 mice were confirmed through breeding with wild-type animals. The tail tips of weanlings (4 weeks old) were cut to obtain genomic DNA using the HotSHOT method [28]. PCR amplifications were performed using Quick Taq HS DyeMix (TOYOBO, Osaka, Japan) with the following conditions: 94 °C for 2 min, followed by 35 cycles of 95 °C for 30 s, 55 °C for 30 s, and 68 °C for 30 s. Heterozygous mice were used in all experiments.

Male NPGM-Cre mice (8 weeks old) were housed individually under standard conditions (25 ± 1 °C under a 12 h light/12 h dark cycle) with ad libitum access to water and either NC (CE-2; CLEA Japan, Tokyo, Japan) or HFD (60% of calories from fat, 7.1% of calories from sucrose; D12492, Research Diets, New Brunswick, NJ, USA).

### 2.2. Production of Plasmid Enabling Cre-Dependent Overexpression of Npgm

The artificial sequence (*Npgm-*P2A-eGFP) designed for the Cre-dependent overexpression of *Npgm* in NPGM-Cre mice was generated using Eurofins Genomics (Tokyo, Japan). To generate pAAV-hSyn-DIO-*Npgm*-P2A-eGFP, pAAV-hSyn-DIO-hM3Dq-mCherry (#44361, Addgene, Watertown, MA, USA) was modified, replacing the coding sequence of hM3Dq-mCherry with the artificial sequence.

### 2.3. Preparation of AAV-Based Vectors

AAV-based vectors were prepared following previous reports [23,29,30] using pAAV-hSyn-DIO-*Npgm*-P2A-eGFP, pAAV-hSyn-DIO-hM3Dq-mCherry, pAAV-EF1α-DIO-mCherry (#50462, addgene), and pAAV-flex-taCasp3-TEVp (#45580, addgene). The AAV-based vectors were stored at −80 °C until use.

### 2.4. Stereotaxic Surgery

AAV injection was performed as described in previous methods [23,29,30]. NPGM-Cre mice were anesthetized using isoflurane. AAVs were bilaterally delivered into the mediobasal hypothalamic region 2.2 mm caudal to the bregma, 0.25 mm lateral to the midline, and 5.8 mm ventral to the skull surface, using a Neuros Syringe (7001 KH; Hamilton, Reno, NV, USA). Next, 0.5 µL of AAV-EF1α-DIO-mCherry (3.2 × 10^9^ genome copies/µL) was delivered into the target regions of NPGM-Cre mice to confirm the neural-specific gene expression. For Cre-dependent overexpression of *Npgm*, 0.5 µL of AAV-hSyn-DIO-*Npgm*-P2A-eGFP (1.8 × 10^9^ genome copies/µL) was injected into the target regions of NPGM-Cre mice. As a control, the same volume of AAV-hSyn-DIO-P2A-eGFP (1.2 × 10^9^ genome copies/µL) was delivered into the target regions of NPGM-Cre mice. Then, 0.5 µL of AAV-hSyn-DIO-hM3Dq-mCherry (7.2 × 10^8^ genome copies/µL) was used for acute/chronic stimulation of NPGM neurons. For the ablation of NPGM-neurons, 0.5 µL of AAV-flex-taCasp3-TEVp (2.2 × 10^10^ genome copies/µL) or AAV-EF1α-DIO-mCherry, as a control, was injected into the target regions of NPGM-Cre mice.

### 2.5. Cre-Dependent Overexpression of Npgm

After AAV injection, NPGM-Cre mice were fed the NC for 28 days. Food intake and body mass were measured at the beginning of the light period (9:00). At the end of this study, mice were decapitated between 13:00 and 15:00. The mediobasal hypothalamus, adipose tissue, and the liver were collected, weighed, and frozen in liquid nitrogen. Blood was simultaneously collected and centrifuged for 15 min at 3000× *g* at 4 °C after incubation for 30 min at 25 ± 1 °C. Plasma was stored at −80 °C. After removing lymph nodes in the iWAT, over 80% of the entire iWAT and eWAT were collected as digestion solutions containing Collagenase I (250 U, Worthington, Lakewood, NJ, USA), DNase I (21.6 U, Worthington), and PBS for flow cytometry.

### 2.6. Stimulation of NPGM Neurons in the Hypothalamus

One week after viral injection, NPGM-Cre mice were fed an HFD for 1 week. Food deprivation was induced at 17:00 in the measurement day. Immediately before the dark period, saline or clozapine-*N*-oxide (CNO) (1 mg/kg) (Tocris Bioscience, Minneapolis, MN, USA) was injected intraperitoneally into NPGM-Cre mice. Simultaneously, re-feeding of an HFD was initiated. Food intake was measured at 1, 2, 4, 6, and 12 h after intraperitoneal (i.p.) injection. The following day, a crossover study was conducted on these mice. Activation of hM3Dq-expressing neurons in these mice was confirmed using immunohistochemistry after an additional CNO injection.

For chronic stimulation of NPGM neurons, NPGM-Cre mice were allowed to recover for 2 weeks with ad libitum access to the NC after stereotaxic surgery. They were then provided drinking water containing CNO (5 mg/kg) to stimulate NPGM neurons via hM3Dq for 2 weeks. Water without CNO was used as the control. Food intake and body mass were measured during the initial 3 days of CNO consumption.

### 2.7. Ablation of NPGM Neurons

Before AAV injection, NPGM-Cre mice were fed an HFD for 8 weeks. The mice were fed HFDs for 56 days after stereotaxic surgery. An oral glucose tolerance test (OGTT) and an insulin tolerance test (ITT) were conducted 42 and 49 days later, respectively. Moreover, 56 days after stereotaxic surgery, each tissue sample was collected as the same procedure in Section 2.5.

### 2.8. Quantitative Reverse Transcriptase PCR (qRT-PCR)

RNA isolation and qRT-PCR were performed as previously reported [23,30,31]. Total RNA was isolated using the TRIzol reagent (Life Technologies, Carlsbad, CA, USA). First-strand cDNA was reverse-transcribed using the PrimeScript RT Reagent Kit with a gDNA Eraser (TAKARA, Shiga, Japan). qRT-PCR was performed with a THUNDERBIRD SYBR qPCR Mix (TOYOBO) under the following conditions: 95 °C for 20 s, followed by 40 cycles of 95 °C for 3 s and 60 °C for 30 s. Data were analyzed using the 2^−ΔΔCt^ method with *β-actin* (*Actb*) [32]. The primer sequences used in this study are listed in Table 1.

### 2.9. Flow Cytometry

The iWAT and the eWAT were minced and shaken at 37 °C for 30 min in a digestion solution. The digested samples were filtered through 300 (pluriSelect Life Science, Leipzig, Germany), 70 (Funakoshi, Tokyo, Japan), and 40 µm (Funakoshi) cell strainers with cation/phenol red-free Hank’s balanced salt solution (HBSS, Thermo Fisher Scientific, Waltham, MA, USA) buffer containing 2% bovine serum albumin (BSA, Sigma-Aldrich, St. Louis, MO, USA), 1 mM EDTA, and 25 mM HEPES (Thermo Fisher Scientific). After centrifugation at 400× *g* for 3 min at 4 °C, red blood cells were lysed with a lysis buffer (Abcam, Cambridge, UK) for 5 min and resuspended in HBSS buffer.

Harvested cells in the stromal vascular fractions were blocked with anti-mouse CD16/CD32 antibodies against Fc receptors (BD Biosciences, San Jose, CA, USA) for 10 min on ice. These cells were incubated for 30 min in an HBSS buffer with BV510 mouse anti-F4/80 (Biolegend, San Diego, CA, USA) or FITC mouse anti-F4/80 (Biolegend), PE mouse anti-CD11c (Biolegend) or FITC mouse anti-CD11c (Biolegend), APC mouse anti-CD206 (Biolegend) or PE-Cy7 mouse anti-CD206 (Biolegend), PerCP/Cy5.5 mouse anti-CD3 (Biolegend) or FITC mouse anti-CD3 (Biolegend), PE-Cy7 mouse anti-CD45 (Biolegend) or PE mouse anti-CD45 (Biolegend), BV421 mouse anti-CD19 (Biolegend) or PE-Cy7 mouse anti-CD19 (Biolegend) on ice. The dead cells were stained with propidium iodide. The stained cells were analyzed using a CytoFLEX S (Beckman Coulter, Brea, CA, USA) or a Cell Sorter MA900 (SONY, Tokyo, Japan). The macrophages were identified as F4/80^+^ cells. The M1 macrophages were identified as F4/80^+^, CD11c^+^, and CD206^−^. The M2 macrophages were identified as F4/80^+^, CD11c^−^, and CD206^+^. The T cells were identified as CD45^+^ and CD3^+^ cells. The B cells were identified as CD45^+^ and CD19^+^ cells. To compare with a negative control, unstained cells were also analyzed for comparison.

### 2.10. Immunohistochemistry

After fixation in 4% paraformaldehyde, cryoprotection, and freezing, the brain tissues were sectioned at a thickness of 20 µm with a cryostat at −20 °C. Immunohistochemistry of free-floating sections was performed as previously described [20,21,23]. The sections were incubated in a blocking buffer (1% BSA, 1% normal donkey serum, and 0.3% Triton X-100 in 10 mM PBS) for 1 h at 25 ± 1 °C before incubation with primary antibodies, including guinea pig anti-NPGM (1:100 or 1:200 dilution), rat anti-GFP (1:50,000 dilution, GF090R; Nacalai Tesque, Kyoto, Japan), goat anti-mCherry (1:500 dilution, AB0040-200; Origene), and rabbit anti-c-fos (1:1000 dilution, sc-52; Santa Cruz Biotechnology, Dallas, TX, USA) overnight at 4 °C. After undergoing three washes with 10 mM PBS, the floating sections were incubated for 1 h at 25 ± 1 °C with secondary antibodies, including Alexa Fluor 488-labeled donkey antibody to rabbit anti-IgG (1:400 or 1:600 dilution, 711-545-152; Jackson ImmunoResearch Laboratories, West Grove, PA, USA), Alexa Fluor 488-labeled donkey antibody to rat anti-IgG (1:500 dilution, 712-545-150; Jackson ImmunoResearch Laboratories), Cy3-labeled donkey antibody to guinea pig anti-IgG (1:400 dilution, 706-165-148; Jackson ImmunoResearch Laboratories), and Alexa Fluor 568-labeled donkey antibody to goat anti-IgG (1:400 dilution, ab175474; Abcam). The immunoreactive labeling was observed using a microscope (Eclipse E600; Nikon, Tokyo, Japan).

### 2.11. Plasma and Hepatic Biochemical Analysis

A GLUCOCARD G+ meter (Arkray, Kyoto, Japan) was used to measure the glucose content of the plasma. The NEFA C-test (Wako Pure Chemical Industries, Osaka, Japan) was used to measure the free fatty acid levels. Finally, the Triglyceride E-Test (Wako Pure Chemical Industries) was used to measure triglyceride levels.

To extract triglycerides from the liver, a previously reported protocol [33] was followed. The livers were homogenized in PBS, and a chloroform–methanol solution (2:1) was added to the homogenates. The samples were centrifuged at 18,000× *g* for 5 min at 4 °C, and the lower layer was collected and evaporated. The extracted lipids were dissolved in 100% isopropanol and hepatic triglyceride levels were measured using the Triglyceride E-Test (Wako Pure Chemical Industries).

### 2.12. OGTT and ITT

The OGTT and the ITT were conducted as previously reported [16,30,34,35,36], 42 and 49 days after stereotaxic surgery, respectively, to induce the ablation of NPGM neurons. Briefly, NPGM-Cre mice were fasted for 16 h for the OGTT or 4 h for the ITT. Using a GLUCOCARD G+ blood glucose meter, blood glucose levels were measured at 0, 15, 30, 60, and 120 min after oral glucose administration for the OGTT (1 g/kg weight) and i.p. injection of insulin for the ITT (0.75 units/kg). A 35 µL blood sample was collected from the tail vein using a heparinized plastic hematocrit tube (Drummond Scientific Company, Broomall, PA, USA), and the plasma was separated through centrifugation at 3000× *g* for 15 min. After centrifugation, the plasma was stored at −80 °C for insulin measurement. The LBIS Insulin-Mouse-U ELISA kit (Shibayagi, Gunma, Japan) was used to measure the insulin levels. The area under the curve (AUC) and inverse AUC for blood glucose were calculated using the linear trapezoidal method for both OGTT and ITT.

### 2.13. Statistical Analysis

A Student’s *t*-test was performed to assess differences between the 2 groups (control and treated mice). To assess the main effects of groups (between control and treated) and time, a two-way, repeated-measures ANOVA was conducted, followed by Sidak’s test for multiple comparisons. Differences at *p* values < 0.05 were considered statistically significant. All results are presented as the mean ± standard error of the mean (SEM).

## 3. Results

### 3.1. Generation of NPGM-Cre Mice

**Figure 1:**
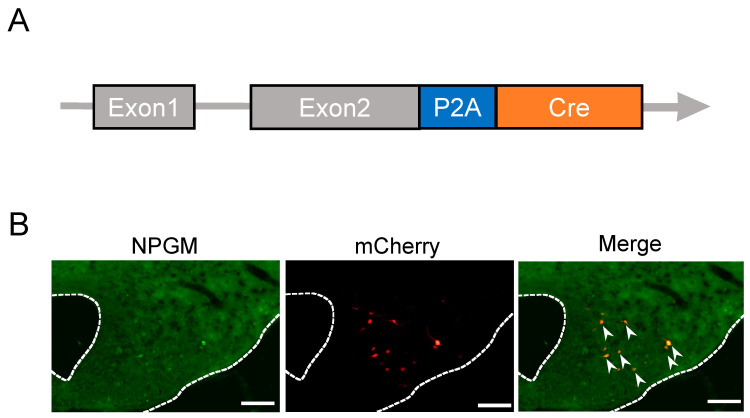
Generation and verification of NPGM-Cre mice. (**A**) Targeted alleles in the NPGM-Cre mice. (**B**) Representative micrographs of the mediobasal hypothalamus at 4 weeks after injection with AAV-EF1α-DIO-mCherry. The arrowheads indicate NPGM-immunoreactive mCherry-expressing neurons. NPGM: neurosecretory protein GM. Scale bars = 100 µm.

To enable a neuron-specific approach to NPGM-expressing cells, we created Cre driver mice using the CRISPR-Cas9 system. The construction of the targeted allele is shown in Figure 1A. The TGA stop codon in exon 2 of *Npgm* was replaced with a 2A self-cleaving peptide and Cre recombinase. To confirm Cre-dependent gene expression in the created mice, we stereotaxically injected AAV-EF1α-DIO-mCherry into the ArcLP. Immunohistochemistry following stereotaxic injection of AAV-EF1α-DIO-mCherry into the ArcLP confirmed the co-localization of NPGM-immunoreactive cells and AAV-derived mCherry (Figure 1B). These results indicate Cre-dependent gene expression in the generated mice, which were named “NPGM-Cre.”

### 3.2. Cre-Dependent Overexpression of Npgm

**Figure 2:**
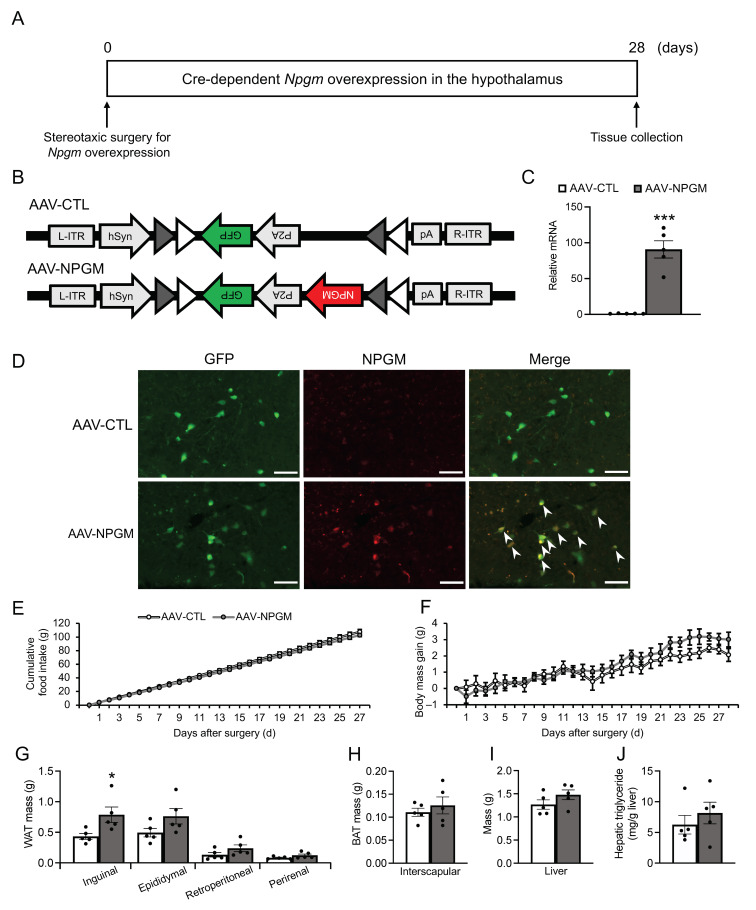
Cre-dependent overexpression of *Npgm* in the hypothalamus of NPGM-Cre mice. (**A**) Experimental procedure. (**B**) Construction of AAV-based vector. (**C**) mRNA expression level of *Npgm* in the mediobasal hypothalamus at the end of *Npgm* overexpression. (**D**) Representative micrograph of the mediobasal hypothalamus at 20 days after injection of AAV-CTL or AAV-NPGM. The arrowheads indicate NPGM-immunoreactive GFP-expressing neurons. (**E**) Cumulative food intake. (**F**) Body mass gain. (**G**) Mass of the inguinal, epididymal, retroperitoneal, and perirenal WAT. (**H**) Mass of the interscapular BAT. (**I**) Mass of the liver. (**J**) Content of hepatic triglycerides. Each value represents the mean ± SEM (*n* = 5). Asterisks indicate statistically significant differences (* *p* < 0.05, *** *p* < 0.005). Differences between groups were assessed using Student’s *t-*test or two-way ANOVA with repeated measures followed by Sidak’s test for multiple comparisons. NPGM: neurosecretory protein GM; AAV-CTL: AAV-based control vector; AAV-NPGM: AAV-based NPGM-precursor gene vector; WAT: white adipose tissue; BAT: brown adipose tissue. Scale bars = 100 µm.

To examine neuron-specific overexpression of *Npgm*, Cre-dependent overexpression of *Npgm* in NPGM-Cre mice was performed via stereotaxic delivery of AAV-hSyn-DIO-*Npgm*-P2A-eGFP (Figure 2A,B). *Npgm* overexpression was confirmed in the ArcLP using qRT-PCR (Student’s *t*-test, df = 8, *t* = 7.430, *p* < 0.005, *n* = 5) and immunohistochemistry (Figure 2C,D). Notably, *Npgm* overexpression had limited effects on cumulative food intake or body mass gain (Figure 2E,F). While the mass of the iWAT was increased due to the overexpression of *Npgm* (Student’s *t*-test, df = 8, *t* = 2.620, *p* < 0.05, *n* = 5), other adipose tissues remained unchanged (Figure 2G,H). In addition to the adipose tissues, apart from the iWAT, the liver mass and the hepatic triglyceride content were unaffected (Figure 2I,J).

**Figure 3:**
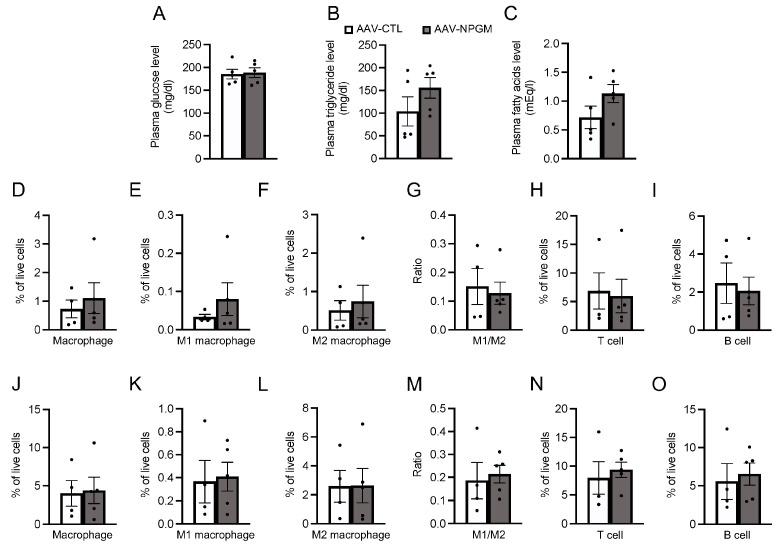
Effects of Cre-dependent overexpression of *Npgm* on blood parameters (**A**–**C**) and the population of immune cells in the iWAT (**D**–**I**) and eWAT (**J**–**O**). Plasma levels of (**A**) glucose, (**B**) triglyceride, and (**C**) free fatty acids. Populations of (**D**) macrophages, (**E**) M1 macrophages, (**F**) M2 macrophages, (**G**) M1/M2, (**H**) T cells, and (**I**) B cells in the iWAT. Populations of (**J**) macrophages, (**K**) M1 macrophages, (**L**) M2 macrophages, (**M**) M1/M2, (**N**) T cells, and (**O**) B cells in the eWAT. Each value represents the mean ± SEM (*n* = 4–5). Differences between groups were assessed using Student’s *t-*test. NPGM: neurosecretory protein GM; AAV-CTL: AAV-based control vector; AAV-NPGM: AAV-based NPGM-precursor gene vector; iWAT: inguinal white adipose tissue; eWAT: epididymal white adipose tissue; M1 macrophages: classically activated macrophages; M2 macrophages: alternatively activated macrophages.

Fat accumulation contributes to hyperglycemia and hyperlipidemia with chronic inflammation [37,38]. To confirm the effects of Cre-dependent *Npgm* overexpression on blood parameters, the blood glucose, triglyceride, and free fatty acids levels were evaluated. Importantly, the blood parameters were not altered due to *Npgm* overexpression (Figure 3A–C). Subsequent examination of immune cell percentages in the iWAT and eWAT revealed no changes (Figure 3D–O). These results suggest that Cre-dependent overexpression of *Npgm* induces fat accumulation without substantial effects on feeding behavior, body mass, blood parameters, or chronic inflammation in adipose tissue.

### 3.3. Acute and Chronic Activation of NPGM Neurons in the Hypothalamus

**Figure 4:**
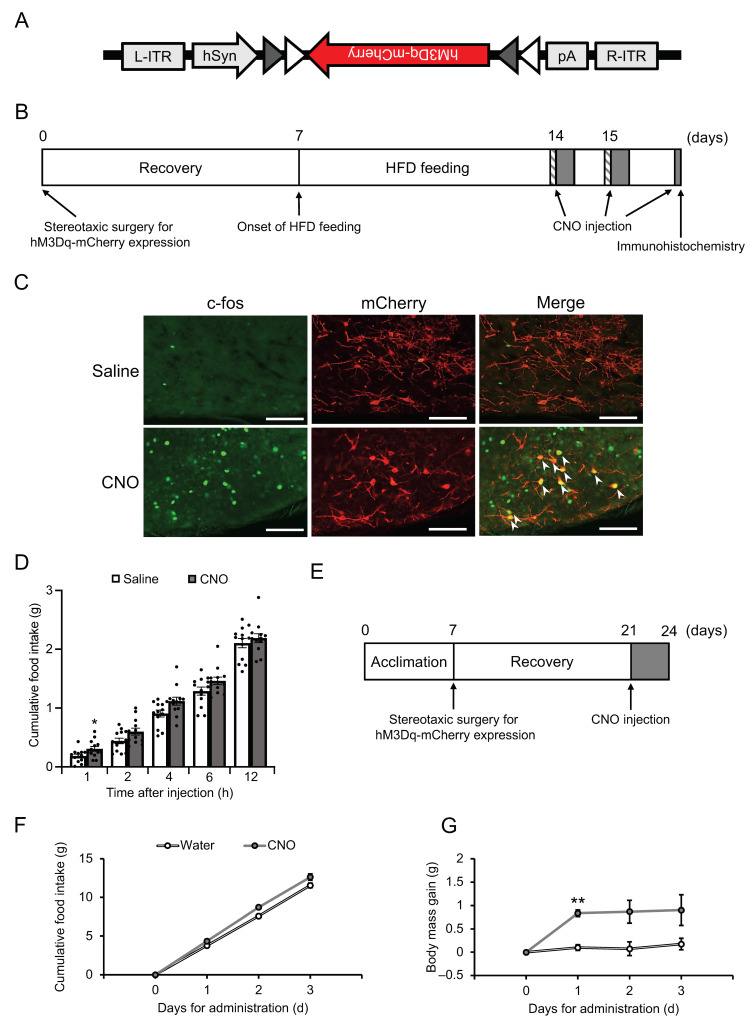
Acute (**B**–**D**) and chronic activation (**E**–**G**) of NPGM neurons in the hypothalamus. (**A**) Construction of AAV-based vector. (**B**) Experimental procedure to acutely activate NPGM neurons in the hypothalamus. Diagonal lines show food deprivation, and the gray zones indicate activation of NPGM neurons. (**C**) Representative micrographs of the mediobasal hypothalamus 90 min after injection of CNO in NPGM-Cre mice expressing hM3Dq. Arrowheads indicate c-fos-immunoreactive mCherry-expressing neurons. (**D**) Cumulative food intake after CNO injection. (**E**) Experimental procedure to chronically activate NPGM neurons in the hypothalamus. The gray zone indicates the activation of NPGM neurons. (**F**) Cumulative food intake after drinking CNO. (**G**) Body mass gain after drinking CNO. Each value represents the mean ± SEM (*n* = 3–13). Asterisks indicate statistically significant differences (* *p* < 0.05, ** *p* < 0.01). Differences between groups were assessed using the Student’s *t-*test or two-way ANOVA with repeated measures followed by Sidak’s test for multiple comparisons. NPGM: neurosecretory protein GM; CNO: clozapine-*N*-oxide. Scale bars = 100 µm.

Considering the established role of hypothalamic neuropeptide-expressing neurons in energy metabolism [11,15,16], NPGM neurons may also be involved in feeding behavior and body mass changes. Acute chemogenetic activation of NPGM neurons was then employed through the bilateral delivery of AAV-hSyn-DIO-hM3Dq-mCherry into the ArcLP (Figure 4A,B). After the i.p. injection of CNO, a ligand specific to hM3Dq, an increase in the number of c-fos/mCherry-double-positive cells was observed through immunostaining (Figure 4C). The CNO-injected mice exhibited an increase in food intake 1 h after injection (paired *t*-test, df = 12, *t* = 2.213, *p* < 0.05, *n* = 13) (Figure 4D). To determine the effect of chronic stimulation of NPGM neurons on body mass changes, testing was then conducted (Figure 4E). CNO was provided in the drinking water. Chronic administration of CNO slightly increased the cumulative food intake (Figure 4F). While body mass gain transiently increased 1 d after CNO administration (two-way, repeated ANOVA, time: *F*_1.639,8.193_ = 5.496, *p* < 0.05, group: *F*_1,5_ = 10.46, *p* < 0.05, interaction: *F*_3,15_ = 3.162, *p* = 0.056; 1 d: Sidak, *p* < 0.01, *n* = 3–4), the effect diminished over time (Figure 4G).

### 3.4. Ablation of NPGM Neurons

**Figure 5:**
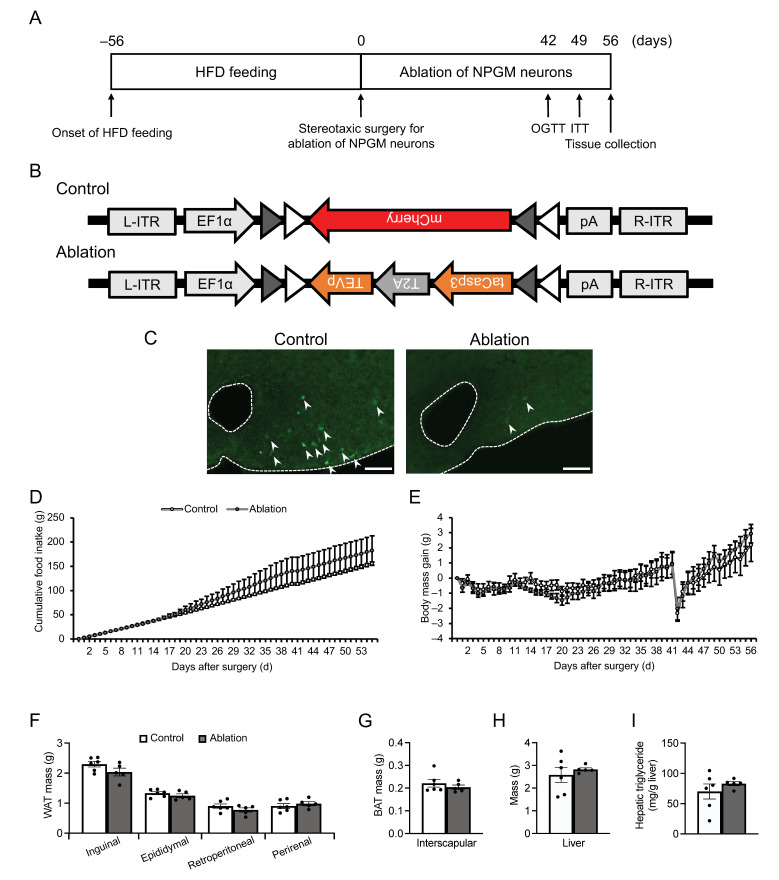
Ablation of NPGM neurons in the hypothalamus of NPGM-Cre mice. (**A**) Experimental procedure of the ablation of NPGM neurons in the hypothalamus. (**B**) Construction of AAV-based vector. (**C**) Representative micrograph of the mediobasal hypothalamus at 4 weeks after injection of AAVs for the control and ablation groups, respectively. The arrowheads indicate NPGM-immunoreactive neurons. (**D**) Cumulative food intake. (**E**) Body mass gain. (**F**) Mass of the inguinal, epididymal, retroperitoneal, and perirenal WAT. (**G**) Mass of the interscapular BAT. (**H**) Mass of the liver. (**I**) Content of hepatic triglyceride. Each value represents the mean ± SEM (*n* = 5–6). Differences between groups were assessed using Student’s *t-*test or two-way ANOVA with repeated measures followed by Sidak’s test for multiple comparisons. NPGM: neurosecretory protein GM; WAT: white adipose tissue; BAT: brown adipose tissue. Scale bars = 100 µm.

To further evaluate the role of NPGM neurons in energy metabolism, NPGM neurons were ablated in HFD-induced obese NPGM-Cre mice using modified caspase 3 (Figure 5A,B). The AAV induces apoptosis in a Cre-dependent manner [39]. Confirmation revealed that fewer NPGM-immunoreactive cells were present than those in control mice (Figure 5C). Unexpectedly, the cumulative food intake and body mass gain were not affected by the disruption in NPGM neurons (Figure 5D,E). In addition to the change in body mass, the mass of the adipose tissues and the liver and the hepatic triglyceride content remained at control levels (Figure 5F–I).

**Figure 6:**
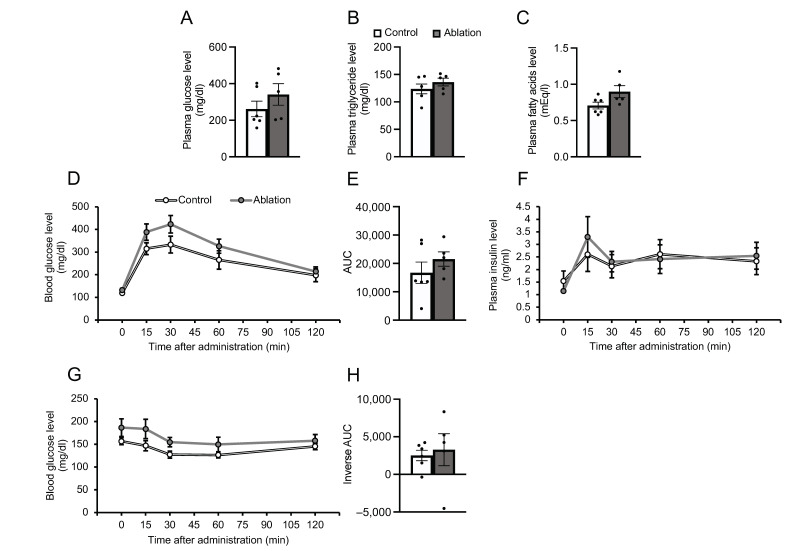
Effects of ablation of NPGM neurons on blood parameters (**A**–**C**) and glucose metabolism (**D**–**H**). Plasma levels of (**A**) glucose, (**B**) triglyceride, and (**C**) free fatty acids. (**D**) Blood level of glucose, (**E**) AUC for blood level of glucose, and (**F**) plasma level of insulin after oral injection of glucose. (**G**) Blood level of glucose and (**H**) AUC for blood level of glucose after i.p. injection of insulin. Each value represents the mean ± SEM (*n* = 5–6). Differences between groups were assessed using Student’s *t-*test. NPGM: neurosecretory protein GM; AUC: area under the curve; i.p.: intraperitoneal.

Subsequently, the effects of NPGM neuron ablation on the glucose/lipid metabolism were confirmed. Blood parameters were unchanged after NPGM neuronal ablation (Figure 6A–C). The OGTT showed a slight tendency toward increased blood glucose levels after glucose injection (not significant, 15 min: Student’s *t*-test, df = 9, *t* = 1.516, *p* = 0.164, *n* = 5–6), independent of insulin secretion (Figure 6D–F). In contrast, elevated blood glucose levels were not observed during the ITT in NPGM-neuron-ablated mice (Figure 6G,H). These results suggest that the ablation of NPGM neurons may exacerbate glucose tolerance.

**Figure 7:**
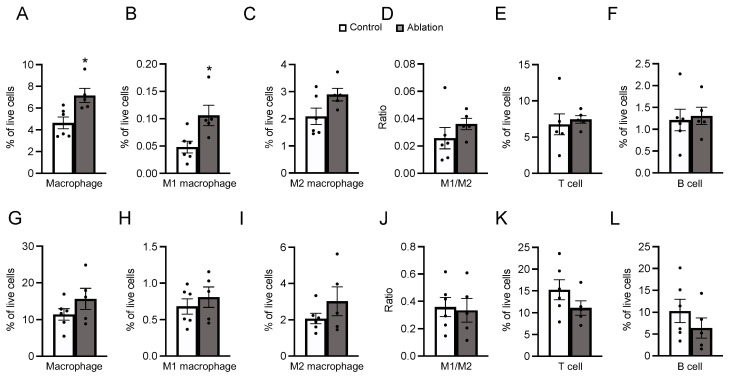
Effects of ablation of NPGM neurons on the population of immune cells in the iWAT (**A**–**F**) and eWAT (**G**–**L**). Populations of (**A**) macrophages, (**B**) M1 macrophages, (**C**) M2 macrophages, (**D**) M1/M2, (**E**) T cells, and (**F**) B cells in the iWAT. Populations of (**G**) macrophages, (**H**) M1 macrophages, (**I**) M2 macrophages, (**J**) M1/M2, (**K**) T cells, and (**L**) B cells in the eWAT. Each value represents the mean ± SEM (*n* = 5–6). Asterisks indicate statistically significant differences (* *p* < 0.05). Differences between groups were assessed using Student’s *t*-test. NPGM: neurosecretory protein GM; iWAT: inguinal white adipose tissue; eWAT: epididymal white adipose tissue; M1 macrophages: classically activated macrophages; M2 macrophages: alternatively activated macrophages.

Glucose intolerance is attributed to chronic inflammation in the adipose tissue [40]. The percentage of immune cells in the iWAT and eWAT was explored. In the iWAT of NPGM-neuron-ablated mice, the percentages of macrophages and M1 macrophages increased (macrophage: Student’s *t*-test, df = 9, *t* = 3.036, *p* < 0.05, *n* = 5–6, M1 macrophage: Student’s *t*-test, df = 9, *t* = 2.813, *p* < 0.05, *n* = 5–6) (Figure 7A,B). Aside from these populations, the percentage of immune cells did not change (Figure 7C–L). The data suggest that NPGM neurons may worsen glucose metabolism by exacerbating inflammatory responses in the iWAT.

## 4. Discussion

The novel hypothalamic small protein NPGL is known to lead to overeating and fat accumulation, resulting in obesity [19,20,21,23]. Compared to the analysis of NPGL, the effects of the paralogous gene NPGM and its expressing neurons on energy metabolism have remained relatively unexplored in mice. In this study, a new Cre driver mouse, NPGM-Cre, was generated, and a neuron-specific approach to NPGM-expressing cells was employed to investigate the relationship between NPGM neurons and energy metabolism. Cre-dependent overexpression of *Npgm* resulted in fat accumulation in the iWAT without an increase in feeding behavior. The activation of NPGM neurons transiently stimulated feeding behavior and body mass gain. Moreover, the ablation of NPGM neurons slightly induced glucose intolerance with the progression of chronic inflammation in the iWAT. In summary, these results suggest that NPGM neurons maintain glucose homeostasis by regulating lipid storage and anti-inflammatory responses.

Although Cre-dependent overexpression of *Npgm* expanded fat depots in the iWAT, the food intake and mRNA expression levels of lipid-metabolism-related factors in the iWAT were equivalent to those in the control. The Arc, in which NPGM neurons are localized, is known to regulate lipid metabolism in the WAT under the regulation of the SNS [41]. Norepinephrine (NE) released from the axon terminus stimulates increased cAMP production via the β-adrenergic receptor [42]. cAMP triggers the phosphorylation of adipose triglyceride lipase (ATGL), hormone-sensitive lipase (HSL), perilipin, and lipolytic enzymes via activation of protein kinase A, resulting in lipolysis [42]. Furthermore, retrograde transsynaptic tracing from the iWAT revealed relatively more labeling in the suprachiasmatic nucleus (SCN), dorsomedial hypothalamus (DMH), and Arc than from the eWAT [41]. Consequently, fat accumulation independent of hyperphagia due to Cre-dependent *Npgm* overexpression may be caused by the suppression of SNS. Owing to the three Cys residues in mammalian NPGM, three patterns are expected for potential disulfide bonds [43]. However, the pattern of endogenous NPGM remains unknown. Given that the translation of overexpressed *Npgm* and the processing of precursor proteins depend on the endogenous regulatory system in NPGM neurons, the overexpressed NPGM in this study can be an appropriate form as well as an intrinsic NPGM. Future studies are required to unravel the intrinsic disulfide patterns and their effects on fat accumulation to further our understanding of endogenous NPGM-related fat accumulation.

Chronic activation of NPGM neurons via hM3Dq transiently stimulated food intake but subsequently abolished this effect. Chronic neural activation by the designer receptors exclusively activated by designer drugs (DREADD) system has provided essential insights into neuroscience [44,45,46,47,48]. However, a recent report has pointed out the possibility of receptor desensitization [49]. Indeed, a previous study suggested receptor desensitization during chronic treatment with ligands and recovery of receptor levels to pretreatment levels during the washout period [49,50]. In addition, a study reported that chronic activation of hM3Dq is regulated by negative feedback from inhibitory presynaptic input [45]. Hence, the transient effects on NPGM neurons may be explained by means of receptor desensitization or negative feedback regulation. Meanwhile, previous research has shown that high-dose CNO (10 mg/kg) was reversely metabolized to clozapine, a dopamine/serotonin antagonist, in mice, and recommended the use of an appropriate control group [51,52]. Considering that the dose of CNO in this study was 5 mg/kg, the reverse metabolism of CNO was not considered. Alternatively, recent studies have applied ion channels for the chronic manipulation of neurons [12,15]. Novel approaches, such as the use of ion channels, offer an elegant means of evaluating the role of NPGM neurons in energy metabolism.

The ablation of NPGM neurons using engineered caspase 3 slightly exacerbated glucose tolerance and infiltrated proinflammatory M1 macrophages in the iWAT (subcutaneous WAT) but not in the eWAT (visceral WAT). Low-grade chronic inflammation caused by macrophages in the adipose tissue is well known to be a trigger of metabolic dysfunction including insulin resistance [5]. A previous study estimated that the percentage of macrophages in the adipose tissues is over 50% in obese mice, although it is not even 10% in lean mice [53]. Macrophages in the adipose tissues are classified as M1 macrophages and M2 macrophages, at least [7]. M1 macrophages promote chronic inflammation through the production of proinflammatory cytokines such as TNF-α and interleukin-6 (IL-6), whereas M2 macrophages inhibit chronic inflammation through the secretion of anti-inflammatory cytokines including interleukin-10 (IL-10) [54,55]. Furthermore, subcutaneous and visceral WATs greatly differ in their contributions to chronic inflammation and metabolic abnormalities [56]. Indeed, previous studies have reported the infiltration of macrophages in the visceral rather than subcutaneous WATs of obese animals [57,58]. Given the possibility of metabolic regulation of the iWAT by NPGM neurons via the SNS, it is reasonable to assume that the lesions of NPGM neurons disrupted immune homeostasis in the iWAT only, resulting in limited glucose intolerance.

Cre-dependent overexpression of *Npgm* evoked fat accumulation in the iWAT without affecting the percentage of immune cells in the WAT. However, ablation of the NPGM neurons maintained the WAT mass and led to the infiltration of M1 macrophages into the iWAT. The phenotypes resulting from neuronal ablation are not necessarily identical to those caused by the pharmacological effects of neuropeptides. Melanin-concentrating hormone (MCH) has been shown to be an orexigenic neuropeptide through i.c.v. injection and analysis in KO mice [59,60]. In contrast, MCH-neuron-ablated mice displayed equivalent feeding behavior compared to the controls, suggesting that the orexigenic effects of MCH were antagonized by the anorexigenic effects of nesfatin-1 and cocaine- and amphetamine-regulated transcript (CART) that are co-expressed neuropeptides in MCH neurons [61,62,63,64]. Recent studies have demonstrated that NPGM is coexpressed with transcripts encoding neuropeptides in the Arc [26,65]. Therefore, the discrepancies in phenotypes between Cre-dependent overexpression of *Npgm* and the ablation of NPGM neurons may be accounted for by complicated processes involving other neurotransmitters.

This study has several limitations. Although Cre-dependent overexpression of *Npgm* led to fat accumulation, we only checked the plasma levels of blood glucose, triglyceride, and free fatty acids. Metabolic abnormalities are closely associated with dyslipidemia including high levels of low-density lipoprotein cholesterol and low levels of high-density lipoprotein cholesterol in obese subjects [66]. Additional measurements focused on serum cholesterol levels are required to understand the effects of NPGM on dyslipidemia. In addition, the present study employed only males. Given that the mRNA expression level of *NPGM* in females of quail is comparable to that in males [67], the sex differences in the contribution of NPGM to energy homeostasis might not be considerable. Further studies using female mice will help us to fully understand the sex differences in the effects of NPGM on energy homeostasis. Moreover, although all experiments in this study were employed using adult mice, the mRNA expression of *NPGM* gradually decreased with development in chicks [24]. Therefore, it is possible that manipulation and ablation of NPGM neurons showed minimal effects on energy homeostasis. Elucidation of the expression of *Npgm* and of the activity of NPGM neurons along with developmental stages in NPGM-Cre mice could lead to an understanding of the physiological function of NPGM and NPGM neurons. Finally, the fasting time before the OGTT and ITT was 16 h and 4 h in the present study, respectively, although a recent study revealed that a long time of food deprivation may lead to gluconeogenesis [68]. On the other hand, given that several recent studies still adopted a long time for fasting [34,36], we consider that the fasting time in these tests is controversial. Further experiments are required to reveal the effects of NPGM and NPGM neurons on gluconeogenesis.

In conclusion, NPGM neurons participate in lipid metabolism and inflammatory responses in the WAT. Whilst the relationship between the hypothalamus and adipose tissue has been widely investigated [16,69], this report will provide a stepping stone for understanding the mechanisms underlying fat accumulation and inflammatory responses in the brain. Future studies, including the chronic activation of NPGM neurons over extended periods, hold the potential to substantially advance our understanding of the intricate mechanisms underlying lipid metabolism and inflammatory response within NPGM neurons.

## Figures and Tables

**Table 1:** Sequences of oligonucleotide primers for qRT-PCR.

Gene	Sense Primer (5′ to 3′)	Antisense Primer (5′ to 3′)
*Npgm*	CTCTCTGACGCTGATAGACC	AGATACTGTAATGCCCAGGA
*Actb*	GGCACCACACCTTCTACAAT	AGGTCTCAAACATGATCTGG
Genotyping 1	CGTTCTGCTGTTCAGTCTCACTG	GATTCCATTCTTCTATGCAACCCAT
Genotyping 2	GCTGATGATCCGAATAACTACCTG	GATTCCATTCTTCTATGCAACCCAT

## Data Availability

The raw data supporting the findings of this manuscript will be made available by the corresponding authors, Y.N. and K.U., to any qualified researchers upon reasonable request.
